# Effect of high-potency cannabis on corpus callosum microstructure

**DOI:** 10.1017/S0033291715002342

**Published:** 2015-11-27

**Authors:** S. Rigucci, T. R. Marques, M. Di Forti, H. Taylor, F. Dell'Acqua, V. Mondelli, S. Bonaccorso, A. Simmons, A. S. David, P. Girardi, C. M. Pariante, R. M. Murray, P. Dazzan

**Affiliations:** 1 Department of Neurosciences, Mental Health and Sensory Organs, Sapienza University of Rome, Rome, Italy; 2 Department of Psychosis Studies, Institute of Psychiatry, Psychology and Neuroscience, King's College London, London, UK; 3 Centre for Neuroimaging Sciences, Institute of Psychiatry, Psychology and Neuroscience, King's College London, London, UK; 4 Department of Psychological Medicine, Institute of Psychiatry, Psychology and Neuroscience, King's College London, London, UK; 5 National Institute for Health Research (NIHR) Mental Health Biomedical Research Centre at South London and Maudsley NHS Foundation Trust and King's College London, London, UK

**Keywords:** Cannabis, corpus callosum, first-episode psychosis, tractography, white matter

## Abstract

**Background:**

The use of cannabis with higher Δ9-tetrahydrocannabinol content has been associated with greater risk, and earlier onset, of psychosis. However, the effect of cannabis potency on brain morphology has never been explored. Here, we investigated whether cannabis potency and pattern of use are associated with changes in corpus callosum (CC) microstructural organization, in patients with first-episode psychosis (FEP) and individuals without psychosis, cannabis users and non-users.

**Method:**

The CC of 56 FEP (37 cannabis users) and 43 individuals without psychosis (22 cannabis users) was virtually dissected and segmented using diffusion tensor imaging tractography. The diffusion index of fractional anisotropy, mean diffusivity (MD), axial diffusivity (AD) and radial diffusivity was calculated for each segment.

**Results:**

Across the whole sample, users of high-potency cannabis had higher total CC MD and higher total CC AD than both low-potency users and those who never used (*p* = 0.005 and *p* = 0.004, respectively). Daily users also had higher total CC MD and higher total CC AD than both occasional users and those who never used (*p* = 0.001 and *p* < 0.001, respectively). However, there was no effect of group (patient/individuals without psychosis) or group x potency interaction for either potency or frequency of use. The within-group analysis showed in fact that the effects of potency and frequency were similar in FEP users and in users without psychosis.

**Conclusions:**

Frequent use of high-potency cannabis is associated with disturbed callosal microstructural organization in individuals with and without psychosis. Since high-potency preparations are now replacing traditional herbal drugs in many European countries, raising awareness about the risks of high-potency cannabis is crucial.

## Introduction

Cannabis use has been associated with an increased risk of subsequent psychosis (Henquet *et al.* 2008; Casadio *et al.* 2011). Our group has previously shown that this risk is greater, and onset occurs earlier, in those individuals who use more frequently and those who use cannabis with higher Δ9-tetrahydrocannabinol (THC) content (high-potency types such as ‘skunk’) (Di Forti *et al.* 2009, 2014). Exploring the role of potency in increasing the risk of psychosis has become particularly important since, over the last decade, modern ‘high-potency’ products (sinsemilla or ‘skunk’) in ‘street cannabis’ have been found to have higher THC (16–22%) and lower cannabidiol (CBD) (<0.1%) content (Potter *et al.* 2008). Interestingly, the THC component of cannabis has been proposed to have a neurotoxic effect on the brain (Gilman *et al.* 2014), while the CBD component has been proposed to be actually neuroprotective (Pertwee, 2008). While the long-term use of cannabis has been associated with alterations in both brain function and morphology (Lorenzetti *et al.* 2010; Schacht *et al.* 2012; Battistella *et al.* 2014), the effect of potency on the brain has never been explored.

THC acts on cannabinoid-1 (CB1) receptors, which, among other effects, modulate a variety of glial cell functions, including oligodendrocytes, and may induce microstructural changes in white matter (WM) (Walter *et al.* 2003). Indeed, oligodendrocyte survival is affected by cannabis exposure during development, with consequent alteration of normal WM development in animals (Molina-Holgado *et al.* 2002). The largest WM tract, the corpus callosum (CC), is of particular interest in humans, since it is very rich in cannabinoid receptors during neurodevelopment. As the CC has a fundamental role in inter-hemispheric connectivity, it is not surprising that this structure has been widely implicated in both psychosis and cannabis-associated behaviours (Arnone *et al.* 2006; Walterfang *et al.* 2008).

Alterations in the microstructural organization of the CC and other WM structures have been reported in patients with psychosis *in vivo* using diffusion tensor imaging (DTI) (Brambilla *et al.* 2005; Kanaan *et al.* 2005; Shergill *et al.* 2007; Cheung *et al.* 2008; Kyriakopoulos *et al.* 2008). While some studies have suggested that alterations are uniformly distributed along the CC, others have suggested that certain segments may be particularly affected. For example, the splenium and the genu seem to be the most affected areas and those that most contribute to the lower CC fractional anisotropy (FA – a measure of the degree of directionality and coherence of WM fibres) observed in individuals with schizophrenia (Buchsbaum *et al.* 2006; Friedman *et al.* 2008; Kubicki *et al.* 2008; Gasparotti *et al.* 2009). However, only three studies have specifically evaluated diffusion microstructural properties of WM in patients with psychosis who were also cannabis users, with inconsistent findings (Peters *et al.* 2009; Dekker *et al.* 2010; James *et al.* 2011). Two studies found that early cannabis use in patients with schizophrenia was associated with increased FA in the splenium of the CC (Dekker *et al.* 2010), and of other tracts such as the uncinate fasciculus, internal capsule and frontal WM (Peters *et al.* 2009). In contrast, the third study found that early cannabis use was associated with lower FA in the brain stem, internal capsule, corona radiata, and superior and inferior longitudinal fasciculi (James *et al.* 2011). These inconsistencies could be due to the use of small samples, the presence of co-morbidities, and, above all, differences in the assessment of cannabis consumption. In fact, no study to date has examined the relationship between potency of cannabis used and CC microstructure.

Interestingly, changes in CC integrity have often been observed in individuals without psychosis but with a history of heavy and long-term cannabis use. These include increase in mean diffusivity (MD – a measure of the average mobility of water molecules, affected by cellular density, extracellular space volume and the overall water content, impairment in axonal connectivity, and reductions in global efficiency), indicating a less efficient and/or slower information transfer across the whole brain (Gruber *et al.* 2005; Arnone *et al.* 2008; Zalesky *et al.* 2012).

Here, we have investigated for the first time the effect of cannabis potency, as well as of frequency and age of first use, on the microstructural organization of the CC using DTI, in a sample of cannabis users and non-users, with and without psychosis. We hypothesized that CC microstructural organization would be particularly affected in individuals who use higher-potency cannabis, independently of frequency and age of first use. We additionally explored whether this effect would be stronger in those individuals with concomitant psychosis, and also investigated which specific segment of the CC, if any, would be altered in relation to cannabis potency.

## Method

### Sample

A total of 56 patients with first-episode psychosis (FEP) were recruited from South East London (UK). Also, 43 individuals without psychosis were recruited from the same geographical area; they were administered the Psychosis Screening Questionnaire (Bebbington *et al.* 1995), and excluded if they reported any psychotic symptom or a history of psychotic illnesses. Exclusion criteria for all subjects included: history of head trauma or injury with loss of consciousness longer than 1 h; current or past organic psychosis; learning disabilities or lack of English fluency (for details, see Di Forti *et al.* 2009, 2014). Ethical approval was obtained from the local ethics committee. After a complete description of the study, written informed consent was obtained. All patients underwent clinical and magnetic resonance imaging (MRI) assessments, as soon as possible after their first contact with services. Individuals without psychosis underwent the same neuroimaging assessment. At the time of the MRI, 48 patients were taking atypical antipsychotics, five were taking typical antipsychotics and three were antipsychotic naive.

### Clinical assessment

International Classification of Diseases (ICD)-10 diagnoses were formulated by qualified psychiatrists using the Operational Criteria Checklist for Psychotic Illness (OPCRIT+) (McGuffin *et al.* 1991), which shows good inter-rater reliability (*κ* = 0.9). The sample included 14 patients with a diagnosis of schizophrenia, 12 acute psychotic disorders, eight schizo-affective disorder, five unspecified non-organic psychosis, 10 bipolar affective disorder and seven severe depressive episode with psychotic symptoms. Severity of psychotic symptoms was assessed with the Positive and Negative Syndrome Scale (PANSS) (Kay *et al.* 1987). Duration of untreated psychosis was quantified as the interval between first onset of psychotic symptoms and first contact with psychiatric services. Psychosis onset was defined using the Nottingham Onset Schedule (Singh *et al.* 2005).

Antipsychotic doses were converted to chlorpromazine equivalents (Woods, 2003), and length of exposure calculated in number of days. Finally, handedness was evaluated with the Annett Hand Preference Questionnaire (Annett, 1970).

### Assessment of cannabis use

A detailed history of illicit drug use (cannabis, stimulants and any other recreational drug) was taken using the Cannabis Experience Questionnaire modified version (Di Forti *et al.* 2009). This allows a detailed assessment of lifetime patterns of cannabis use, including: frequency and duration of use, the specific type of cannabis used and age at first use. The measures of exposure to cannabis use included in the analyses were: (*a*) lifetime history of cannabis use (had the subject ever used cannabis at any point in the lifetime: no = 0; yes = 1); (*b*) lifetime frequency of cannabis use [the frequency that characterized the subject's most consistent pattern of use: none = 0; at weekends or less frequently (occasional)=1; every day (daily)=2]; (*c*) type of cannabis used [the potency of cannabis used that characterized the subject's most consistent pattern of use: no = 1; low-potency (hash-like)=2; high-potency (skunk-like)=3]; (*d*) age at first use (the age when the subject started to use cannabis regularly: prior to age 15 years = 0; above age 15 years = 1). Evaluating frequency of use with this approach estimates the pattern that represents most of the subject's use during his/her period of cannabis use, and distinguishes those individuals who have been mostly regular users from those who have been more occasional users. Pattern of cannabis use in patients and individuals without psychosis is detailed in Table 1.

**Table 1.** Demographic and clinical characteristics and patterns of cannabis use

	First-episode psychosis patients (*n* = 56)	Individuals without psychosis (*n* = 43)	Test statistics^a^
Mean age, years (s.d.)	28.8 (8)	27.4 (10)	df = 93, *t* = 0.85, n.s.
Gender, *n*			*χ*^2^ = 2.66, n.s.
Male	31	22	
Female	25	21	
Mean duration of education, years (s.d.)	13.4 (3.5)	14.7 (3.0)	df = 93, *t* = −1.86, *p* = 0.06
Ethnicity, *n* (%)			
White Caucasian	19 (37)	18 (50)	*χ*^2^ = 2.87, n.s.
Black Caribbean	9 (17)	7 (20)	
Black African	14 (27)	8 (22)	
Other	10 (19)	3 (8)	
Diagnosis by ICD-10, *n*		–	–
Schizophrenia	14		
Acute psychotic disorder	12		
Schizo-affective disorder	8		
Unspecified non-organic psychosis	5		
Bipolar affective disorder	10		
Depressive episode with psychotic symptoms	7		
Mean total antipsychotic dose, CPZ equivalents (s.d.)	7830.7 (8838.0)	–	–
Mean DUP, days (s.d.)	116 (182)	–	–
Mean PANSS score (s.d.)			
Total	52.7 (13.5)	–	–
Positive	12.2 (15.3)		
Negative	13.1 (15.6)		
General	27.6 (17.6)		
Ever used cannabis, *n* (%)	37 (70)	22 (52)	df = 1, *χ*^2^ = 4.0, *p* = 0.04
Mean duration of cannabis use, years (s.d.)^b^	7.6 (9)	7.2 (5)	df = 43, *t* = 1.0, n.s.
Type of cannabis used, *n* (%)^c^			
No cannabis use	16 (32)	22 (55)	df = 2, *χ*^2^ = 5.6, *p* = 0.06
Low potency (hash-like)	11 (22)	6 (15)	
High potency (skunk-like)	23 (46)	12 (30)	
Age at first use, *n* (%)			
<15 years	12 (32.4)	6 (27.3)	df = 1, *χ*^2^ = 0.2, n.s.
>15 years	25 (67.6)	16 (72.7)	
Frequency of use, *n* (%)^d^			
Occasional	11 (30)	11 (50)	df = 2, *χ*^2^ = 5.3, *p* = 0.07
Daily	25 (70)	11 (50)	
Other drugs, *n* (%)^e^	22 (64.7)	12 (35.3)	df = 2, *χ*^2^ = 5.9, *p* = 0.05
Mean TIV, ml (s.d.)	1716.5 (201.8)	1726.7 (175.8)	df = 90, *t* = −0.52, n.s.
Mean WMV, ml (s.d.)	449.8 (51.0)	458.8 (55.8)	df = 90, *t* = −0.81, n.s.
Mean GMV, ml (s.d)	751 (100.7)	775.5 (83.1)	df = 90, *t* = −1.26, n.s.
Mean CSF, ml (s.d.)	513.1 (121.2)	486.8 (109.1)	df = 90, *t* = 1.1, n.s.

s.d., Standard deviation; df, degrees of freedom; n.s., non-significant; ICD, International Classification of Diseases; CPZ, chlorpromazine; DUP, duration of untreated psychosis; PANSS, Positive and Negative Syndrome Scale; TIV, total intracranial volume; WMV, white matter volume; GMV, grey matter volume; CSF, cerebrospinal fluid.

a Only *p*'s < 0.1 are reported.

b Lack of details for one patient and one control.

c Lack of details for six patients and three controls.

d Lack of details for one patient.

e Lack of details for 16 patients and 11 controls.

### DTI

#### Image acquisition

Data were acquired on a 3.0-Tesla, using a GE Signa-HDx system running software release 14M5, with actively shielded magnetic field gradients (maximum amplitude 40 mT/m). A body coil was used for radiofrequency transmission, and an eight-channel head coil for signal reception, allowing a parallel imaging (ASSET) speed up factor of two. Each volume was acquired using a multi-slice peripherally gated doubly refocused spin echo planar imaging (EPI) sequence, optimized for precise measurement of the diffusion-tensor in parenchyma, from 60 contiguous near-axial slice locations with isotropic (2.4  ×  2.4  ×  2.4 mm) voxels. Echo time was 104.5 ms and effective repetition time varied between 12 and 20 R-R intervals. Acquisition was gated to the cardiac cycle using a peripheral gating device. Maximum diffusion weighting was 1300 s/mm^2^, and at each slice location, four images were acquired with no diffusion gradients applied, together with 32 diffusion-weighted images with gradient directions uniformly distributed in space. An in-house automated analysis technique assessed the quality of EPI data.

#### Image processing

The raw diffusion dataset was then submitted to a full quality-control check, where all *b* = 0 values and diffusion-weighted volumes were visually inspected using the light-box function available inside fslview for any image corruption, motion artifacts and signal drop-out effects. Any dataset showing significant head movements (>1 cm) or more than two motion artifacts in different volumes on the same slice were removed from the study. Diffusion data were processed using ExploreDTI (Leemans *et al.* 2009). Data were first pre-processed correcting for eddy current distortions and head motion. For each subject the b-matrix was then reoriented to provide a more accurate estimate of tensor orientations. The diffusion tensor was estimated using a non-linear least square approach (Jones *et al.* 2002), with FA, MD, radial diffusivity (RD) and axial diffusivity (AD) calculated from the diffusion tensor. We report on RD and AD measures as they can provide information on the nature of changes present in WM tracts. For example, RD has been suggested to be a marker of reduction in myelin content, representing an index of axonal demyelination, while AD is indicative of axonal damage (Beaulieu *et al.* 2002).

#### Tractography analysis

Tractography was started in all brain voxels with FA > 0.2. Streamlines were propagated using Euler integration applying a b-spline interpolation of the diffusion tensor field (Basser *et al.* 1994), and the tractography algorithm step size of 0.5 mm. When FA was <0.2, or when the angle between two consecutive tractography steps was larger than 30 ×, tractography stopped. Finally, FA, MD, RD and AD indices were measured along the tract using TrackVis v0.4.3 software (Wang *et al.* 2007).

#### CC dissection

TrackVis (http://www.trackvis.org) was used for virtual dissection of the CC. In order to dissect this tract, a single region of interest (ROI) was used as previously described (Catani *et al*. 2002; Catani & Thiebaut de Schotten, 2008). To include the entirety of the CC and avoid false-negative fibres, the single ROI was drawn large-sized around the CC on a mid-sagittal slice, following the anatomy of the different segments of this tract and according to *a priori* anatomical knowledge (Yasmin *et al.* 2009). All false-positive components were removed using one or more NOT-ROI, which is an ROI used for the exclusion of fibres considered not to be part of the CC.

#### CC segmentation

**Fig. 1.**
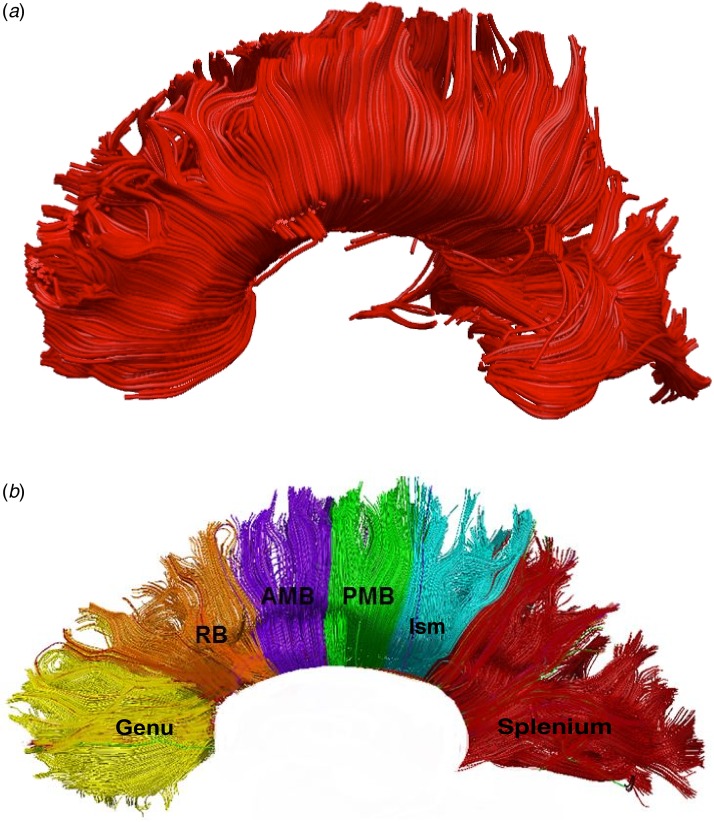
Corpus callosum tract: whole (*a*) and segmented (*b*). Regions of interest as defined according to Witelson (1989) subdivisions. RB, Rostral body; AMB, anterior mid-body; PMB, posterior mid-body; Ism, isthmus. For a colour figure, see the online version.

The subsections of the CC were defined according to the geometrical instructions given by Witelson (1989). The maximal length of the CC was taken as the line joining the most anterior and posterior point of the callosum. Perpendiculars to this axis were drawn at specific arithmetic divisions resulting in callosal segments, which are shown in Fig. 1*b*. The length of CC could then be divided into the fractions described by Witelson (1989), using a new ROI for each subsection. These include: genu, rostral body, anterior mid-body, posterior mid-body, isthmus and splenium (Fig. 1). The ROI for each subsection spans the proportion of the total CC length assigned to it by the Witelson subdivisions (for example, the splenium ROI was one-fifth of the anterior–posterior length of the ROI), and perpendicular height (inferior to superior) at least equal to the corresponding part of the CC ROI, forming a rectangle.

### Data analysis

Sociodemographic characteristics were examined with *t* tests or *χ*^2^ as appropriate. We first examined differences in CC microstructure between FEP patients and individuals without psychosis using multivariate analyses of covariance (MANCOVAs), with FA, MD, RD and AD values of the whole CC as dependent variables, group as fixed factor (patients *v.* individuals without psychosis), and age and gender as covariates of no interest. To examine the effect of cannabis on CC microstructure (for total CC and its subregions) across the whole sample, the same analyses were performed with potency of cannabis (no use, low potency, high potency), frequency of use (never, occasional, daily), cumulative effect of potency/frequency (daily/high-potency users, daily/low-potency users, never used/used weekly) and age at first use (<or >15 years) separately entered as fixed factors. Finally, these analyses where repeated on an exploratory basis separately for patients with psychosis and individuals without psychosis.

## Results

Patients and individuals without psychosis were similar in terms of age, gender and ethnicity, although patients had, as expected, a lower level of education than individuals without psychosis (*t* = 1.86, *p* = 0.06) (Table 1). Patients were more likely to have ever used cannabis than individuals without psychosis. However, there was no significant difference between patients and individuals without psychosis for years of cannabis use, age at first use, type of cannabis use, frequency of use and potency of cannabis used. There was also no significant difference between groups for alcohol use (*χ*^2^ = 0.12, *p* = 0.7) or alcohol intake in terms of units per week (*χ*^2^ = 4.6, *p* = 0.2). Patterns of cannabis use did not significantly differ across diagnoses (*χ*^2^ = 24.8, *p* = 0.08 for frequency of use; *χ*^2^ = 22.4, *p* = 0.1 for potency; *χ*^2^ = 7.1, *p* = 0.3 for age at first use; and *χ*^2^ = 20.2, *p* = 0.2 for the combined effect of frequency and potency).

### CC microstructural organization

Patients showed a significantly lower total CC FA (*F*_1,93_ = 4.1, *p* = 0.04) than individuals without psychosis. Patients also had higher, albeit non-significantly, total CC MD values than individuals without psychosis (*F*_1,93_ = 2.3, *p* = 0.09). Furthermore, patients had higher total CC RD and AD values than individuals without psychosis, although this difference was not statistically significant (*F*_1,93_ = 1.76, *p* > 0.05 and *F*_1,93_ = 1.78, *p* > 0.05, respectively).

### CC microstructural organization and potency of cannabis use

When we explored the effect of cannabis potency across the whole group (patients and individuals without psychosis), we observed a significant effect on total CC MD (*F*_2,82_ = 5.7, *p* = 0.005), with high-potency users showing significantly higher MD than both low-potency users and those who never used, who in contrast had similar MD values (Table 2). There was no effect of group (patient/individuals without psychosis) (*F* = 1.2, n.s.) or group x potency interaction (*F* = 0.2, n.s.) on MD values. Likewise, none of the covariates had a significant effect (all *p* > 0.05). The analyses on the CC subsections showed that, compared with both low-potency users and those who never used, the users of high-potency cannabis had higher MD of the splenium (*F*_2,80_ = 4.5, *p* = 0.01) and of the genu (*F*_2,80_ = 4.4, *p* = 0.02).

**Table 2.** Corpus callosum microstructural integrity and patterns of cannabis use (potency, frequency and age at first use) across the sample as a whole, and in the two samples of patients with first-episode psychosis and individuals without psychosis

Potency of cannabis use				Frequency of cannabis use				Age at first use			
Corpus callosum measure	Never used	Low potency	High potency	Statistics^a^	Never used	Occasional users	Daily users	Statistics^a^	<15 years old	>15 years old	Statistics^a^
Mean FA (s.d.):											
Whole sample	0.576 (0.01)	0.574 (0.01)	0.573 (0.01)	*F*_2,82_ = 1.8, n.s.	0.576 (0.01)	0.573 (0.01)	0.572 (0.01)	*F*_2,87_ = 0.6, n.s.	0.573 (0.01)	0.574 (0.01)	*F*_1,41_ = 0.01, n.s.
Patients with psychosis	0.572 (0.01)	0.572 (0.01)	0.572 (0.02)	*F*_2,45_ = 0.39, n.s.	0.575 (0.01)	0.572 (0.01)	0.571 (0.01)	*F*_2,48_ = 0.9, n.s.	0.571 (0.01)	0.572 (0.02)	*F*_1,23_ = 0.1, n.s.
Individuals without psychosis	0.582 (0.01)	0.574 (0.01)	0.574 (0.01)	*F*_2,35_ = 2.4, n.s.	0.578 (0.01)	0.576 (0.01)	0.573 (0.01)	*F*_2,37_ = 0.5, n.s.	0.577 (0.01)	0.575 (0.01)	*F*_1,18_ = 0.4, n.s.
Mean MD, × 10^−3^ mm^2^/s (s.d.)											
Whole sample	0.791 (0.02)	0.794 (0.02)	0.807 (0.02)	*F*_2,82_ = 5.7, *p* = 0.005^b^	0.791 (0.01)	0.792 (0.02)	0.808 (0.01)	*F*_2,87_ = 7.5, *p* = 0.001^c^	0.805 (0.02)	0.801 (0.02)	*F*_1,41_ = 0.5, n.s.
Patients with psychosis	0.794 (0.02)	0.798 (0.01)	0.808 (0.02)	*F*_2,45_ = 1.86, n.s.	0.795 (0.02)	0.796 (0.01)	0.809 (0.02)	*F*_2,48_ = 2.87, *p* = 0.06^d^	0.809 (0.02)	0.802 (0.01)	*F*_1,23_ = 0.38, n.s.
Individuals without psychosis	0.788 (0.02)	0.789 (0.02)	0.810 (0.01)	*F*_2,35_ = 4.32, *p* = 0.02^e^	0.786 (0.02)	0.790 (0.02)	0.807 (0.02)	*F*_2,37_ = 4.83, *p* = 0.01^f^	0.811 (0.01)	0.791 (0.02)	*F*_1,18_ = 3.64, *p* = 0.07^d^
Mean RD, × 10^−3^ mm^2^/s (s.d.)											
Whole sample	0.491 (0.01)	0.492 (0.02)	0.502 (0.02)	*F*_2,82_ = 3.4, *p* = 0.04^d^	0.488 (0.02)	0.492 (0.02)	0.502 (0.02)	*F*_2,87_ = 4.3, *p* = 0.02^d^	0.499 (0.02)	0.497 (0.02)	*F*_1,41_ = 0.1, n.s.
Patients with psychosis	0.494 (0.02)	0.496 (0.02)	0.503 (0.02)	*F*_2,45_ = 1.33, n.s.	0.495 (0.02)	0.492 (0.01)	0.504 (0.02)	*F*_2,48_ = 2.3 , n.s.	0.499 (0.02)	0.504 (0.02)	*F*_1,23_ = 0.2, n.s.
Individuals without psychosis	0.489 (0.01)	0.481 (0.01)	0.499 (0.01)	*F*_2,35_ = 2.88, *p* = 0.07	0.483 (0.01)	0.490 (0.01)	0.498 (0.01)	*F*_2,37_ = 2.5, n.s.	0.500 (0.01)	0.487 (0.01)	*F*_1,18_ = 2.4, n.s.
Mean AD, × 10^−3^ mm^2^/s (s.d.)											
Whole sample	1.39 (0.02)	1.40 (0.03)	1.41 (0.03)	*F*_2,82_ = 6.05, *p* = 0.004^g^	1.39 (0.03)	1.40 (0.03)	1.42 (0.03)	*F*_2,87_ = 8.9, *p* < 0.001^h^	1.41 (0.03)	1.40 (0.03)	*F*_1,41_ = 0.8, n.s.
Patients with psychosis	1.39 (0.03)	1.40 (0.02)	1.41 (0.02)	*F*_2,45_ = 1.85, n.s.	1.39 (0.02)	1.40 (0.02)	1.41 (0.03)	*F*_2,48_ = 2.1, n.s.	1.42 (0.03)	1.40 (0.02)	*F*_1,23_ = 0.5, n.s.
Individuals without psychosis	1.38 (0.03)	1.39 (0.04)	1.42 (0.03)	*F*_2,35_ = 4.2, *p* = 0.02^i^	1.38 (0.03)	1.39 (0.02)	1.42 (0.03)	*F*_2,37_ = 5.7, *p* = 0.007^j^	1.43 (0.04)	1.39 (0.03)	*F*_1,18_ = 2.8, *p* = 0.4

FA, Fractional anisotropy; s.d., standard deviation; n.s., non-significant; MD, mean diffusivity; RD, radial diffusivity; AD, axial diffusivity.

a Only *p*'s < 0.1 are reported. *Post-hoc* comparisons are given in notes b to j, all Bonferroni corrected.

b High-potency users had higher corpus callosum MD than those who never used (*p* = 0.004).

c Daily users had higher corpus callosum MD than those who never used (*p* = 0.002), and those who used weekly or less (*p* = 0.004).

d Did not survive Bonferroni correction.

e High-potency users had higher corpus callosum MD than those who never used (*p* = 0.02).

f Daily users had higher corpus callosum MD than those who never used (*p* = 0.02), and those who used weekly or less (*p* = 0.03).

g High-potency users had higher corpus callosum AD than those who never used (*p* = 0.002).

h Daily users had higher corpus callosum AD than those who never used (*p* = 0.001), and those who used weekly or less (*p* = 0.006).

i High-potency users had higher corpus callosum AD than those who never used (*p* = 0.02).

j Users had higher corpus callosum AD than those who never used (*p* = 0.008), and those who used weekly or less (*p* = 0.03).

High-potency users also showed significantly higher AD (*F*_2,82_ = 6.05, *p* = 0.004), and higher RD (*F*_2.82_ = 3.4, *p* = 0.04), than both low-potency users and those who never used (Table 2). There was no effect of group (patient/individuals without psychosis) (*F* = 0.1, n.s.) or group x potency interaction (*F* = 1.3, n.s.) on AD values. The exploratory analysis of the CC subsections showed that, compared with both low-potency users and those who never used, users of high-potency cannabis had significantly higher AD, at trend level in the genu (*F*_2,82_ = 2.7, *p* = 0.06) of the CC. Finally, there was no effect of potency on FA values.

For completion, we investigated the effect of cannabis potency separately in patients and in individuals without psychosis (Table 2). In patients, high-potency users showed higher mean total CC MD and AD than both low-potency users and those who never used, albeit the difference was not statistically significant (*F*_2,45_ = 1.86, *p* = 0.16; and *F*_2,45_ = 1.85, *p* = 0.16, respectively). Also, among individuals without psychosis high-potency users showed significantly higher total CC MD, higher CC AD, and at trend level CC RD, than both low-potency users and those who never used. Finally, there was no effect of potency on FA values in both groups. Comparisons of the CC subsections are presented in the online Supplementary material.

### CC microstructural organization and frequency of cannabis use

Across the whole group, we found a significant effect of frequency of use on total CC MD (*F*_2,87_ = 7.5, *p* = 0.001), with daily users having significantly higher MD than both occasional users and those who never used. There was no effect of group (*F* = 1.8, n.s.) or group x frequency interaction (*F* = 0.2, n.s.) (Table 2). Likewise, none of the covariates showed a significant effect on these MD differences (all *p* > 0.05). The exploratory analysis of the CC subsections showed that the daily users, compared with both occasional users and those who never used, had higher MD in the splenium (*F*_2.87_ = 11.6, *p* < 0.001) and the genu (*F*_2,87_ = 4.9, *p* = 0.01).

We observed no effect of frequency of use on FA values. However, there was a significant effect of frequency on total CC AD (*F*_2,87_ = 8.9, *p* < 0.001) and on total CC RD (*F*_2,87_ = 4,3, *p* = 0.02) values, with daily users having a significantly higher AD and RD values than both occasional users and those who never used. There was no effect of group (patient/individuals without psychosis) (*F* = 0.7, n.s.; *F* = 2.3, n.s., for AD and RD, respectively) or group x potency interaction (*F* = 2.0, n.s.; *F* = 0.1, n.s., for AD and RD, respectively) on these values. The analysis of the CC subsections showed that, compared with both occasional users and those who never used, the daily users had higher AD in the genu (*F*_2,87_ = 3.93, *p* = 0.03) of the CC.

For completion, we investigated the effect of cannabis frequency separately in patients and individuals without psychosis (Table 2). In patients, every-day users showed higher mean total CC MD and total CC AD than both occasional users and those who never used, although the difference was at trend level for MD (*F*_2,48_ = 2.87, *p* = 0.06) and not statistically significant for AD (*F*_2,48_ = 2.1, *p* = 0.1). Also, among individuals without psychosis, every-day users showed significantly higher total CC MD and total CC AD than both occasional users and those who never used. Finally, there was no effect of frequency in FA and RD. Comparisons of the CC subsections are presented in the online Supplementary material.

### CC microstructural organization and the cumulative effect of potency and frequency of cannabis use

Since a considerable number of subjects who used high-potency cannabis were also daily users, and both high potency and daily frequency were significantly associated with altered CC integrity, we conducted an additional MANCOVA using a cumulative score for each individual, to capture the effect of frequency and potency combined (Table 3). We compared: (i) subjects who used high-potency cannabis on a daily basis, *v.* those who (ii) used low-potency cannabis on a daily basis, and *v.* those who (iii) never used or only used weekly. This analysis showed that the daily/high-potency users had a significantly higher total CC MD (*F*_2,82_ = 7.3, *p* = 0.001), CC AD (*F*_2,82_ = 8.8, *p* < 0.001) and CC RD (*F*_2,82_ = 3.7, *p* = 0.03) than those who never used/used weekly, while the daily/low-potency users had CC MD values intermediate between the two but were not significantly different from either. This suggests that the effect of frequency is particularly marked when high-potency compounds are used.

**Table 3.** Corpus callosum microstructural integrity: cumulative effect of frequency and potency of cannabis use across the sample as a whole, and in the two samples of patients with first-episode psychosis and individuals without psychosis

Corpus callosum measure	Cumulative effect of frequency and potency			
	Never used or used weekly	Every day low potency	Every day high potency	Statistics^a^
Mean FA (s.d.)				
Whole sample	0.574 (0.01)	0.574 (0.01)	0.573 (0.01)	*F*_2,82_ = 0.7, n.s.
Patients with psychosis	0.573 (0.01)	0.572 (0.01)	0.570 (0.01)	*F*_2,45_ = 0.7, n.s.
Individuals without psychosis	0.588 (0.01)	0.576 (0.02)	0.574 (0.01)	*F*_2,35_ = 1.32, n.s.
Mean MD, × 10^−3^ mm^2^/s (s.d.)				
Whole sample	0.792 (0.02)	0.798 (0.02)	0.810 (0.02)	*F*_2,82_ = 7.3, *p* = 0.001^b^
Patients with psychosis	0.794 (0.02)	0.807 (0.02)	0.809 (0.02)	*F*_2,45_ = 2.8, *p* = 0.07^c^
Individuals without psychosis	0.771 (0.01)	0.790 (0.01)	0.810 (0.01)	*F*_2,35_ = 6.1, *p* = 0.005^d^
Mean RD, × 10^−3^ mm^2^/s (s.d.)				
Whole sample	0.491 (0.01)	0.495 (0.02)	0.502 (0.02)	*F*_2,82_ = 3.7, *p* = 0.03^e^
Patients with psychosis	0.494 (0.02)	0.504 (0.02)	0.504 (0.03)	*F*_2,45_ = 1.9, n.s.
Individuals without psychosis	0.469 (0.01)	0.489 (0.01)	0.499 (0.01)	*F*_2,35_ = 3.6, *p* = 0.04^f^
Mean AD, × 10^−3^ mm^2^/s (s.d.)				
Whole sample	1.39 (0.03)	1.40 (0.03)	1.42 (0.03)	*F*_2,82_ = 8.8, *p* ⩽ 0.001^g^
Patients with psychosis	1.39 (0.02)	1.41 (0.03)	1.42 (0.02)	*F*_2,45_ = 2.8, *p* = 0.07^c^
Individuals without psychosis	1.38 (0.03)	1.39 (0.04)	1.43 (0.03)	*F*_2,35_ = 6.05, *p* = 0.006^h^

FA, Fractional anisotropy; s.d., standard deviation; n.s., non-significant; MD, mean diffusivity; RD, radial diffusivity; AD, axial diffusivity.

a Only *p*'s < 0.1 are reported. *Post-hoc* comparisons are given in notes b to h, all Bonferroni corrected.

b Every day high-potency users had higher corpus callosum MD than those who never used or used weekly (*p* = 0.001), and those who used every day low-potency cannabis (*p* = 0.09).

c Did not retain statistical significance.

d Every-day high-potency users had higher corpus callosum MD than those who never used or used weekly (*p* = 0.013), and those who used every day low-potency cannabis (*p* = 0.044).

e High-potency users had higher corpus callosum RD than those who never used or used weekly (*p* = 0.04).

f Did not retain statistical significance.

g High-potency users had higher corpus callosum AD than those who never used or used weekly (*p* < 0.001).

h Every-day high-potency users had higher corpus callosum AD than those who never used or used weekly (*p* = 0.006).

The exploratory analysis of the CC subsections showed that the daily/high-potency users had a significantly higher MD than those who used low-potency cannabis daily and those who never used/used weekly in the splenium (*F*_2,80_ = 8.7, *p* < 0.001) and the genu (*F*_2,80_ = 3.9, *p* = 0.03) of the CC; and higher AD in the genu (*F*_2,82_ = 4.2, *p* = 0.02).

For completion, we investigated the combined effect of cannabis frequency and potency separately in patients and individuals without psychosis (Table 3). In patients, daily/high-potency users showed higher mean total CC MD and total CC AD than those who used low-potency cannabis daily and those who never used/used weekly, although the difference was not statistically significant. Also, among individuals without psychosis daily/high-potency users had a significantly higher total CC MD, total CC AD and total CC RD than both those who used low-potency cannabis daily and those who never used/used weekly. Finally there was no effect of the combined effect of cannabis frequency and potency in FA. Comparisons of the CC subsections are presented in the online Supplementary material.

### CC microstructural organization and age at first use

Finally, we evaluated the effect of age at first use on CC integrity across the whole group, and found no significant differences in any CC metrics between early (<15 years) and late cannabis users (>15 years) (*F*_1,41_ = 0.1, *p* = 0.5) (Table 2).

For completion, we investigated the effect of age at first use separately in patients and individuals without psychosis (Table 2). In patients, early-onset users showed higher mean total CC MD and total CC AD than those who started using cannabis later, although the difference was not statistically significant. Also, among individuals without psychosis, early-onset users had a significantly higher total CC MD and total CC AD, than those who started later, although this was not significant. Comparisons of the CC subsections are presented in the online Supplementary material.

## Discussion

To our knowledge, this is the first study to investigate the effect of cannabis potency and frequency on CC WM microstructural organization, both in patients experiencing their first episode of psychosis and in cannabis users without psychosis. Our main finding, which is in line with our main hypothesis, is that frequent use of high-potency cannabis is significantly associated with altered callosal microstructural integrity. Furthermore, our results suggest that this particularly occurs in the most posterior part of the CC, including the splenium and the posterior mid-body. Interestingly, these alterations were similar in users with and without a psychotic disorder.

Our findings support a role for frequent use of high-potency cannabis in altered CC microstructure, suggesting that callosal integrity may be particularly sensitive to high THC concentration. CB1 receptors, on which THC acts, have a known effect on oligodendrocyte development (myelin initiation, deposition, compaction and maintenance) (Davis *et al.* 2003). Hence, chronic or early exposure to high-THC cannabis preparations, compared with those with low THC, may alter WM through a down-regulation of CB1 receptors. This may result in apoptosis of oligodendrocyte progenitors during WM development (Walter *et al.* 2003).

Although cannabis effects on WM have also been related to use that occurs early, when WM is still developing and cannabis receptors are abundant, we found no difference in WM integrity between individuals who started younger or older than 15 years of age. Still, considering that all our high-potency participants started using in their teens, and that WM continues to develop post-adolescence, it is possible that early adulthood remains a period of vulnerability. Indeed, one of the CC areas that we found to be most affected by frequent high-potency cannabis use was the splenium, which completes maturation only in young adulthood, and later than other callosal parts, thus remaining particularly susceptible to toxic effects of cannabis (De Bellis *et al.* 2008). The splenium and the posterior mid-body, which we also found particularly affected, also contain motor fibres (Zarei *et al.* 2006), and their alteration may contribute to a dysfunction of sensorimotor circuits, resulting in sensory perception alterations, impaired sensorimotor gating and hallucinations, all of which have been associated with cannabis abuse (Heng *et al.* 2011).

While one should be cautious about interpreting DTI measures in terms of the pathological process that underlies microstructural changes, it is interesting that, similarly to other studies, we found that cannabis potency and frequency were associated with an increase in MD, but with no changes in FA. MD is a non-specific measure of integrity, and alterations in this measure can result from changes in intra- or extra-cellular space, including extra-cellular oedema, and therefore be temporary and reversible (Bosch *et al.* 2012). Increases in MD are also observed in pathologies accompanied by neuropil reduction and may reflect demyelination or axonal loss (Selemon *et al.* 1999). Cannabis frequency and potency were also associated with an increase in AD and, at trend level, RD. It is possible that neurobiological changes such as fibre reorganization, glial alteration and even axonal degeneration induce water to diffuse in unanticipated directions and therefore increase measures such as AD (Beaulieu *et al.* 2002). The concordance of changes in tensor metrics, with increases in MD, AD and RD, can also lead to proportional non-significant changes in FA, as the ones seen in our study and similar to those observed in other neurological disorders such as Alzheimer's disease (Acosta-Cabronero *et al.* 2010).

We were somewhat surprised to see that differences in callosal integrity in relation to cannabis potency were larger in the individuals without psychosis than in the patients. Comparison with other structural neuroimaging studies of cannabis use in FEP patients is difficult as only three studies to date have used DTI and none has examined patterns of cannabis use. Dekker *et al*. (2010) found reduced callosal FA in a small sample of eight cannabis-naive patients with schizophrenia compared with 10 early-user patients, and no morphological differences between early-onset and late-onset cannabis users with schizophrenia. Unfortunately, the study did not report MD or AD values. In contrast, James *et al*. (2011) found that early cannabis use in adolescent-onset schizophrenia was associated with lower FA in the brain stem, internal capsule, corona radiata, and superior and inferior longitudinal fasciculi. Finally, a recent study in FEP patients found no brain-wide differences in grey matter or WM between lifetime heavy and light users, or non-users (Haller *et al.* 2013). Several methodological differences may explain these inconsistencies. For example, most studies used small samples, and subjects varied in age range and diagnosis, with most including only patients with schizophrenia rather than all psychoses. Also, the methods used to examine WM differ across studies. These factors potentially affect all neuroimaging investigations and make it difficult to extrapolate whether differences in findings are due to sample characteristics or methods used. Still, it is possible that the alterations in WM microstructure we detected in our early, high-potency cannabis-user patients would become even more significant if a larger sample is examined. Of note, we did not find differences in the proportion of cannabis users and the related patterns of use across diagnostic groups. This is an important clinical issue and a study with a larger sample size would allow a more specific evaluation of the role of diagnosis in relation to pattern of cannabis use and brain structure in psychosis.

Our data go further than previous evidence and suggest that cannabis potency and frequency affect the CC in individuals with and without psychosis, and possibly reflect a subtle and general effect rather than altered neuronal integrity. This is consistent with evidence of callosal alterations in non-psychotic long-term and heavy cannabis users (Arnone *et al.* 2008; Zalesky *et al.* 2012). Overall, the finding is even more interesting when we consider that on direct comparison, our patients had significantly lower callosal FA values than individuals without psychosis, suggesting that: (*a*) our patients have alterations similar to those previously reported in psychosis samples (Lener *et al.* 2015); and (*b*) that this was apparent even though our individuals without psychosis included cannabis users.

This study has a number of strengths. We have evaluated the role of cannabis potency in relation to brain structure for the first time. Furthermore, we have used a sample larger than those used in previous studies, and evaluated users both with and without psychosis. This has allowed us to provide data on the effects of cannabis potency and pattern of use independently of the presence of psychosis. In addition, all our patients were at their first psychotic episode. Therefore, cannabis use had occurred prior to (or around) illness onset, and not as a consequence of the illness. Also, patients were not exposed to long-term pharmacological treatment, making it unlikely that WM alterations were due to antipsychotic medications. Finally, we used a reliable and comprehensive fibre-tracking method for the evaluation of the CC, which has additionally provided details on the integrity of its subsections.

Although our sample is one of the largest in which WM and cannabis use have been evaluated, the number of subjects using low-potency cannabis was relatively small. This may actually reflect the shift that has occurred in the UK towards use of more high-potency cannabis, which also reassures us that participants were more likely to admit to their use. The lack of objective measures of cannabis use is another important limitation. However, other studies that have used self-report measures have also shown an association with brain structural alterations (Yücel *et al.* 2008; Cousijn *et al.* 2012). The fact that many of the individuals without psychosis admitted to their cannabis use also gives us confidence that participants were honest and open about their pattern of use. Nevertheless, in a random sample of 56 cases from the original sample, we carried out a urine drug screen to test the reliability of data on current use (up to 4 weeks prior to the assessment). Of the 56 cases tested, 34 had reported they were not current users; 32 of these (94%) had a negative urinary drug screening; only two tested positive (Di Forti *et al.* 2012). In addition, there is published evidence indicating that asking patients with psychosis and individuals from the general population about their use of cannabis is, at least in some situations, more accurate than, or as reliable as, urine or blood testing which can only provide information on recent use (Hjorthøj *et al.* 2011; Freeman *et al.* 2014). Finally, the accuracy of the neuroimaging approach we used is contingent on its reliability, and tensorial tractography models such as those used in this study fail to map multiple fibre orientations in one voxel, and therefore may fail to map the fibres of the CC lateral to the crossing with the cortico-spinal tract. Tractography nevertheless uses information from a larger part of the tract than either voxel-based or tract-based spatial statistics (TBSS) methods of analysis and may therefore be preferable (Dell'Acqua *et al.* 2013).

This study provides the first report that WM disarray is greater among heavy users of high-potency cannabis, than in occasional or low-potency users, and that this is independent of the presence of a psychotic disorder. Unfortunately, high-potency cannabis is replacing traditional herbal cannabis preparations in many European countries. Raising awareness about the risks of high-potency cannabis abuse seems therefore crucial. It will be extremely important that future studies evaluating the effects of cannabis use on brain structure and function include a careful assessment of cannabis potency.

## Supplementary material

For supplementary material accompanying this paper visit http://dx.doi.org/10.1017/S0033291715002342. click here to view supplementary material

## References

[ref1] Acosta-Cabronero J, Williams GB, Pengas G, Nestor PJ (2010). Absolute diffusivities define the landscape of white matter degeneration in Alzheimer's disease. Brain 133, 529–539. 19914928 10.1093/brain/awp257

[ref2] Annett M (1970). A classification of hand preference by association analysis. British Journal of Psychology 61, 303–321. 5457503 10.1111/j.2044-8295.1970.tb01248.x

[ref3] Arnone D, Abou-Saleh MT, Barrick TR (2006). Diffusion tensor imaging of the corpus callosum in addiction. Neuropsychobiology 54, 107–113. 17108711 10.1159/000096992

[ref4] Arnone D, Barrick TR, Chengappa S, Mackaya CE, Clark CA, and Abou-Saleh MT (2008). Corpus callosum damage in heavy marijuana use: preliminary evidence from diffusion tensor tractography and tract-based spatial statistics. NeuroImage 41, 1067–1074. 18424082 10.1016/j.neuroimage.2008.02.064

[ref6] Basser PJ, Matiello J, Le Bihan D (1994). Estimation of the effective self-diffusion tensor from the NMR spin echo. Journal of Magnetic Resonance, Series B 103, 247–254. 8019776 10.1006/jmrb.1994.1037

[ref7] Battistella G, Fornari E, Annoni JM, Chtioui H, Dao K, Fabritius M, Favrat B, Mall JF, Maeder P, Giroud C (2014). Long-term effect of cannabis on brain structure. Neuropsychopharmacology 39, 2041–2048. 24633558 10.1038/npp.2014.67 PMC4104335

[ref8] Beaulieu JM, Kriz J, Julien JP (2002). Induction of peripherin expression in subsets of brain neurons after lesion injury or cerebral ischemia. Brain Research 946, 153–161. 12137917 10.1016/s0006-8993(02)02830-5

[ref9] Bebbington PE, Nayani T (1995). The Psychosis Screening Questionnaire. International Journal of Methodology in Psychiatry Research 5, 11–20.

[ref10] Bosch B, Arenaza-Urquijo EM, Rami L, Sala-Llonch R, Junqué C (2012). Multiple DTI index analysis in normal aging, amnestic MCI and AD. Relationship with neuropsychological performance. Neurobiology of Aging 33, 61–74. 20371138 10.1016/j.neurobiolaging.2010.02.004

[ref11] Brambilla P, Cerini R, Gasparini A, Versace A, Andreone N, Vittorini E (2005). Investigation of corpus callosum in schizophrenia with diffusion imaging. Schizophrenia Research 79, 201–210. 15953707 10.1016/j.schres.2005.04.012

[ref12] Buchsbaum MS, Friedman J, Buchsbaum BR, Chu KW, Hazlett EA (2006). Diffusion tensor imaging in schizophrenia. Biological Psychiatry 60, 1181–1187. 16893533 10.1016/j.biopsych.2005.11.028

[ref13] Casadio P, Fernandes C, Murray RM, Di Forti M (2011). Cannabis use in young people: the risk for schizophrenia. Neuroscience and Biobehavioral Reviews 35, 1779–1787. 21530584 10.1016/j.neubiorev.2011.04.007

[ref15] Catani M, Howard RJ, Pajevic S, Jones DK (2002). Virtual *in vivo* interactive dissection of white matter fasciculi in the human brain. NeuroImage 17, 77–94. 12482069 10.1006/nimg.2002.1136

[ref16] Catani M, Thiebaut de Schotten M (2008). A diffusion tensor imaging tractography atlas for virtual *in vivo* dissections. Cortex 44, 1105–1132. 18619589 10.1016/j.cortex.2008.05.004

[ref17] Cheung V, Cheung C, McAlonan GM, Deng Y, Wong JG (2008). A diffusion tensor imaging study of structural dysconnectivity in never-medicated, first-episode schizophrenia. Psychological Medicine 38, 877–885. 17949516 10.1017/S0033291707001808

[ref18] Cousijn J, Wiers RW, Ridderinkhof KR, van den Brink W, Veltman DJ, Goudriaan AE (2012). Grey matter alterations associated with cannabis use: results of a VBM study in heavy cannabis users and healthy controls. NeuroImage 59, 3845–3851. 21982932 10.1016/j.neuroimage.2011.09.046

[ref19] Davis KL, Stewart DG, Friedman JI, Buchsbaum M, Harvey PD, Hof PR, Buxbaum J, Haroutunian V (2003). White matter changes in schizophrenia: evidence for myelin-related dysfunction. Archives of General Psychiatry 60, 443–456. 12742865 10.1001/archpsyc.60.5.443

[ref20] De Bellis MD, Van Voorhees E, Hooper SR, Gibler N, Nelson L (2008). Diffusion tensor measures of the corpus callosum in adolescents with adolescent onset alcohol use disorders. Alcohol Clinical Experimental Research 32, 395–404. 10.1111/j.1530-0277.2007.00603.x PMC3566638 18241319

[ref21] Dekker N, Schmitz N, Peters BD, van Amelsvoort TA, Linszen DH, de Haan L (2010). Cannabis use and callosal white matter structure and integrity in recent-onset schizophrenia. Psychiatry Research 181, 51–56. 19962862 10.1016/j.pscychresns.2009.06.003

[ref22] Dell'Acqua F, Simmons A, Williams SC, Catani M (2013). Can spherical deconvolution provide more information than fiber orientations? Hindrance modulated orientational anisotropy, a true-tract specific index to characterize white matter diffusion. Human Brain Mapping 34, 2464–2483. 22488973 10.1002/hbm.22080 PMC6870506

[ref23] Di Forti M, Iyegbe C, Sallis H, Kolliakou A, Falcone MA, Paparelli A, Sirianni M, La Cascia C, Stilo SA, Marques TR, Handley R, Mondelli V, Dazzan P, Pariante C, David AS, Morgan C, Powell J, Murray RM (2012). Confirmation that the AKT1 (rs2494732) genotype influences the risk of psychosis in cannabis users. Biological Psychiatry 72, 811–816. 22831980 10.1016/j.biopsych.2012.06.020

[ref24] Di Forti M, Morgan C, Dazzan P, Pariante C, Mondelli V, Marques TR, Handley R, Luzi S, Russo M, Paparelli A, Butt A, Stilo SA, Wiffen B, Powell J, Murray RM (2009). High-potency cannabis and the risk of psychosis. British Journal of Psychiatry 195, 488–491. 19949195 10.1192/bjp.bp.109.064220 PMC2801827

[ref25] Di Forti M, Sallis H, Allegri F, Trotta A, Ferraro L, Stilo SA, Marconi A, La Cascia C, Reis Marques T, Pariante C, Dazzan P, Mondelli V, Paparelli A, Kolliakou A, Prata D, Gaughran F, David AS, Morgan C, Stahl D, Khondoker M, MacCabe JH, Murray RM (2014). Daily use, especially of high-potency cannabis, drives the earlier onset of psychosis in cannabis users. Schizophrenia Bulletin 6, 1509–1517. 24345517 10.1093/schbul/sbt181 PMC4193693

[ref26] Freeman TP, Morgan CJ, Hindocha C, Schafer G, Das RK, Curran HV (2014). Just say ‘know’: how do cannabinoid concentrations influence users’ estimates of cannabis potency and the amount they roll in joints? Addiction 109, 1686–1694. 24894801 10.1111/add.12634

[ref27] Friedman JI, Tang C, Carpenter D, Buchsbaum M, Schmeidler J (2008). Diffusion tensor imaging findings in first-episode and chronic schizophrenia patients. American Journal of Psychiatry 165, 1024–1032. 18558643 10.1176/appi.ajp.2008.07101640

[ref28] Gasparotti R, Valsecchi P, Carletti F, Galluzzo A, Liserre R (2009). Reduced fractional anisotropy of corpus callosum in first-contact, antipsychotic drug-naive patients with schizophrenia. Schizophrenia Research 108, 41–48. 19103476 10.1016/j.schres.2008.11.015

[ref29] Gilman JM, Kuster JK, Lee S, Lee MJ, Kim BW, Makris N, van der Kouwe A, Blood AJ, Breiter HC (2014). Cannabis use is quantitatively associated with nucleus accumbens and amygdala abnormalities in young adult recreational users. Journal of Neuroscience 34, 529–538. 10.1523/JNEUROSCI.4745-13.2014 PMC3988409 24741043

[ref30] Gruber SA, Yurgelun-Todd DA (2005). Neuroimaging of marijuana smokers during inhibitory processing: a pilot investigation. Cognitive Brain Research 23, 107–118. 15795138 10.1016/j.cogbrainres.2005.02.016

[ref31] Haller S, Curtis L, Badan M, Bessero S, Albom M, Chantraine F, Alimenti A, Lovblad KO, Giannakopoulos P, Merlo M (2013). Combined grey matter VBM and white matter TBSS analysis in young first episode psychosis patients with and without cannabis consumption. Brain Topography 26, 641–647. 23604786 10.1007/s10548-013-0288-8

[ref32] Heng L, Beverley JA, Steiner H, Tseng KY (2011). Differential developmental trajectories for CB1 cannabinoid receptor expression in limbic/associative and sensorimotor cortical areas. Synapse 65, 278–286. 20687106 10.1002/syn.20844 PMC2978763

[ref33] Henquet C, Van Os J (2008). The coherence of the evidence linking cannabis with psychosis. Psychological Medicine 38, 461–462. 18298878 10.1017/S0033291707002279

[ref34] Hjorthøj CR, Fohlmann A, Larsen A-M, Arendt M and Nordentoft M (2011). Correlations and agreement between delta-9-tetrahydrocannabinol (THC) in blood plasma and timeline follow-back (TLFB)-assisted self-reported use of cannabis of patients with cannabis use disorder and psychotic illness attending the CapOpus randomized clinical trial. Addiction 107, 1123–1131. 22151583 10.1111/j.1360-0443.2011.03757.x

[ref35] James A, Hough M, James S, Winmill L, Burge L, Nijhawan S, Matthews PM, Zarei M (2011). Greater white and grey matter changes associated with early cannabis use in adolescent-onset schizophrenia (AOS). Schizophrenia Research 128, 91–97. 21388791 10.1016/j.schres.2011.02.014

[ref36] Jones DK, Griffin LD, Alexander DC, Catani M, Horsfield MA, Howard R, Williams SC (2002). Spatial normalization and averaging of diffusion tensor MRI sets. NeuroImage 17, 592–617. 12377137

[ref37] Kanaan RA, Kim JS, Kaufmann WE, Pearlson GD, Barker GJ, McGuire PK (2005). Diffusion tensor imaging in schizophrenia. Biological Psychiatry 58, 921–929. 16043134 10.1016/j.biopsych.2005.05.015

[ref38] Kay SR, Fiszbein A, Opler LA (1987). The Positive and Negative Syndrome Scale (PANSS) for schizophrenia. Schizophrenia Bulletin 13, 261–276. 3616518 10.1093/schbul/13.2.261

[ref39] Kubicki M, Styner M, Bouix S, Gerig G, Markant D (2008). Reduced interhemispheric connectivity in schizophrenia-tractography based segmentation of the corpus callosum. Schizophrenia Research 106, 125–131. 18829262 10.1016/j.schres.2008.08.027 PMC2630535

[ref40] Kyriakopoulos M, Vyas NS, Barker GJ, Chitnis XA, Frangou S (2008). A diffusion tensor imaging study of white matter in early-onset schizophrenia. Biological Psychiatry 63, 519–523. 17662964 10.1016/j.biopsych.2007.05.021

[ref41] Leemans A, Jones DK (2009). The B-matrix must be rotated when correcting for subject motion in DTI data. Magnetic Resonance Medicine 61, 1336–1349. 10.1002/mrm.21890 19319973

[ref42] Lener MS, Wong E, Tang CY, Byne W, Goldstein KE, Blair NJ, Haznedar MM, New AS, Chemerinski E, Chu KW, Rimsky LS, Siever LJ, Koenigsberg HW, Hazlett EA (2015). White matter abnormalities in schizophrenia and schizotypal personality disorder. Schizophrenia Bulletin 41, 300–310. 24962608 10.1093/schbul/sbu093 PMC4266294

[ref43] Lorenzetti V, Lubman DI, Whittle S, Solowj N, Yucel M (2010). Structural MRI findings in long-term cannabis users: what do we know? Substance Use and Misuse 45, 1787–1808. 20590400 10.3109/10826084.2010.482443

[ref44] McGuffin P, Farmer A, Harvey I (1991). A polydiagnostic application of operational criteria in studies of psychotic illness. Development and reliability of the OPCRIT system. Archives of General Psychiatry 48, 764–770. 1883262 10.1001/archpsyc.1991.01810320088015

[ref45] Molina-Holgado E, Vela JM, Arévalo-Martín A, Almazán G, Molina-Holgado F, Borrell J, Guaza C (2002). Cannabinoids promote oligodendrocyte progenitor survival: involvement of cannabinoid receptors and phosphatidylinositol-3 kinase/Akt signaling. Journal of Neurosciences 15, 9742–9753. 10.1523/JNEUROSCI.22-22-09742.2002 PMC6757831 12427829

[ref47] Pertwee RG (2008). The diverse CB1 and CB2 receptor pharmacology of three plant cannabinoids: delta9-tetrahydrocannabinol, cannabidiol and delta9-tetrahydrocannabivarin. British Journal of Pharmacology 153, 199–215. 17828291 10.1038/sj.bjp.0707442 PMC2219532

[ref48] Peters BD, de Haan L, Vlieger EJ, Majoie CB, den Heeten GJ, Linszen DH (2009). Recent-onset schizophrenia and adolescent cannabis use: MRI evidence for structural hyperconnectivity? Psychopharmacology Bulletin 42, 75–88. 19629024

[ref49] Potter DJ, Clark P, Brown MB (2008). Potency of delta 9-THC and other cannabinoids in cannabis in England in 2005: implications for psychoactivity and pharmacology. Journal of Forensic Sciences 53, 90–94. 18279244 10.1111/j.1556-4029.2007.00603.x

[ref50] Schacht JP, Hutchison KE, Filbey FM (2012). Associations between cannabinoid receptor-1 (CNR1) variation and hippocampus and amygdala volumes in heavy cannabis users. Neuropsychopharmacology 11, 2368–2376. 22669173 10.1038/npp.2012.92 PMC3442352

[ref51] Selemon LD, Goldman-Rakic PS (1999). The reduced neuropil hypothesis: a circuit based model of schizophrenia. Biological Psychiatry 45, 17–25. 9894571 10.1016/s0006-3223(98)00281-9

[ref52] Shergill SS, Kanaan RA, Chitnis XA, O'Daly O, Jones DK (2007). A diffusion tensor imaging study of fasciculi in schizophrenia. American Journal of Psychiatry 164, 467–473. 17329472 10.1176/ajp.2007.164.3.467

[ref53] Singh SP, Cooper JE, Fisher HL, Tarrant CJ, Lloyd T, Banjo J, Corfe S, Jones P (2005). Determining the chronology and components of psychosis onset: the Nottingham Onset Schedule (NOS). Schizophrenia Research 80, 117–130. 15978778 10.1016/j.schres.2005.04.018

[ref54] Walter L, Franklin A, Witting A, Wade C, Xie Y, Kunos G, Mackie K, Stella N (2003). Nonpsychotropic cannabinoid receptors regulate microglial cell migration. Journal of Neurosciences 23, 1398–1405. 10.1523/JNEUROSCI.23-04-01398.2003 PMC6742252 12598628

[ref55] Walterfang M, Wood AG, Reutens DC, Wood SJ, Chen J, Velakoulis D, McGorry PD, Pantelis C (2008). Morphology of the corpus callosum at different stages of schizophrenia: cross-sectional study in first-episode and chronic illness. British Journal of Psychiatry 192, 429–434. 18515892 10.1192/bjp.bp.107.041251

[ref56] Wang R, Benner T, Sorensen AG, Wedeen VJ (2007). Diffusion Toolkit: a software package for diffusion imaging data processing and tractography. Proceedings of the International Society for Magnetic Resonance in Medicine 15, 3720.

[ref57] Witelson SF (1989). Hand and sex differences in the isthmus and genu of the human corpus callosum: a postmortem morphological study. Brain 112, 799–835. 2731030 10.1093/brain/112.3.799

[ref58] Woods SW (2003). Chlorpromazine equivalent doses for the newer atypical antipsychotics. Journal of Clinical Psychiatry 64, 663–667. 12823080 10.4088/jcp.v64n0607

[ref59] Yasmin H, Aoki S, Abe O, Nakata Y, Hayashi N, Masutani Y, Goto M, Ohtomo K (2009). Tract-specific analysis of white matter pathways in healthy subjects: a pilot study using diffusion tensor MRI. Neuroradiology 51, 831–840. 19662389 10.1007/s00234-009-0580-1

[ref60] Yücel M, Solowij N, Respondek C, Whittle S, Fornito A, Pantelis C, Lubman DI (2008). Regional brain abnormalities associated with long-term heavy cannabis use. Archives of General Psychiatry 65, 694–701. 18519827 10.1001/archpsyc.65.6.694

[ref61] Zalesky A, Solowij N, Yücel M, Lubman DI, Takagi M, Harding IH, Lorenzetti V, Wang R, Searle K, Pantelis C, Seal M (2012). Effect of long-term cannabis use on axonal fibre connectivity. Brain 135, 2245–2255. 22669080 10.1093/brain/aws136

[ref62] Zarei M, Johansen-Berg H, Smith S, Ciccarelli O, Thompson AJ (2006). Functional anatomy of interhemispheric cortical connections in the human brain. Journal of Anatomy 209, 311–320. 16928200 10.1111/j.1469-7580.2006.00615.x PMC2100336

