# Supplementary material for: Effect of high-potency cannabis on corpus callosum microstructure
Source: Psychol Med. 2015 Nov 27;46(4):841–54. doi: 10.1017/S0033291715002342 (PMC4754829; doi:10.1017/S0033291715002342)
Supplement: Supplementary file 1 [file S0033291715002342sup001.doc]

**Supplementary material**

**Cannabis potency and integrity of CC subsections in patients with psychosis and in individuals without psychosis**

In patients, high potency cannabis users had higher AD, at trend level, in the Splenium [F(2,43)= 2.4; *p*=0.09] of the CC than both low-potency users and those who never used. No differences across groups were observed in the MD of the CC subsections.

In individuals without psychosis, high-potency users had higher MD of the Splenium [F(2,35)= 3.19; p=0.05], and a trend level of the Posterior Mid-Body [F(2,35)= 2.7; p=0.08] and Genu [F(2,35)= 3.06; p=0.06] than both low-potency users and those who never used. They also had higher AD of the Posterior Mid-Body [F(5,58)= 3.80; *p*=0.03] and, at trend level, of the Rostral Body [F(2,35)= 3.04; *p*=0.06] of the CC than both low-potency users and those who never used. No differences across groups were observed in AD values of the CC subsections.

**Cannabis frequency and integrity of CC subsections in patients with psychosis and in individuals without psychosis**

In patients, daily users had higher MD in the Splenium (F(2,48)= 5.57; p=0.007), Rostral Body (F(2,48)= 3.39; p=0.04) and a trend level in the Genu (F(2,48)= 2.93; p=0.06) of the CC compared to both occasional users and those who never used. No differences across groups were observed in AD of the CC subsections.

In individuals without psychosis, daily users had higher MD in the Splenium (F(2,37)= 9.37; p=0.001), Isthmus (F(2,37)= 4.01; p=0.026) and at trend level in the Posterior Mid-Body (F(5,63)= 2.37; p=0.08) of the CC compared to both occasional users and those who never used. No differences across groups were observed in AD of the CC subsections.

**Cumulative effect of cannabis potency and frequency and integrity of CC subsections in patients with psychosis and in individuals without psychosis**

In patients, daily/high potency users had higher MD in the Splenium (F(2,43)= 3.68; p=0.03), and in the Genu (F(2,43)= 3.38; p=0.04) of the CC compared to both daily-low potency users, and those who never used/used weekly. No differences were observed in AD of the CC subsections.

In individuals without psychosis, daily/high potency users had higher MD in the Splenium (F(2,35)= 8.06; p=0.001), Isthmus (F(2,35)= 6.12; p=0.005) and the Posterior Mid-Body (F(2,35)= 3.07; p=0.059) of the CC compared to both daily-low potency users and those who never used/used weekly. Furthermore, the daily/high potency users had higher AD in the Genu (F(2,35)= 3.2; p=0.05), Posterior Mid-Body (F(2,35)= 3.38; p=0.04), and at trend level, in the Isthmus (F(2,35)= 2.87; p=0.07) compared to both the daily-low potency users and those who never used/used weekly. No differences were observed across groups in RD of the CC subsections.

**Age at first cannabis use and integrity of CC subsections in patients with psychosis and in individuals without psychosis**

In patients and individuals without psychosis there were no significant differences in any WM values of CC subregions (all p>0.05) between those who started before the age of 15 years and those who started later than 15 years of age.
